# Circulating microRNAs and Outcome in Patients with Acute Heart Failure

**DOI:** 10.1371/journal.pone.0142237

**Published:** 2015-11-18

**Authors:** Marie-France Seronde, Mélanie Vausort, Etienne Gayat, Emeline Goretti, Leong L. Ng, Iain B. Squire, Nicolas Vodovar, Malha Sadoune, Jane-Lise Samuel, Thomas Thum, Alain Cohen Solal, Said Laribi, Patrick Plaisance, Daniel R. Wagner, Alexandre Mebazaa, Yvan Devaux, GREAT network

**Affiliations:** 1 Department of Cardiology, EA3920, University Hospital, Besançon, France; 2 UMRS 942 Inserm, University Paris Diderot, Paris, France; 3 Laboratory of Cardiovascular Research, Centre de Recherche Public de la Santé, Luxembourg, Luxembourg; 4 Department of Anesthesiology and Critical Care Saint Louis—Lariboisière Hospital, Paris, France; 5 University of Leicester and National Institute for Health Research, Leicester Cardiovascular Biomedical Research Unit, Glenfield Hospital, Leicester, United Kingdom; 6 Institute of Molecular and Translational Therapeutic Strategies (IMTTS), Hannover, Germany; 7 Department of Cardiology, Lariboisière Hospital, Paris, France; 8 Department of Emergency Medicine, Lariboisière Hospital, Paris, France; Texas A& M University Health Science Center, UNITED STATES

## Abstract

**Background:**

The biomarker value of circulating microRNAs (miRNAs) has been extensively addressed in patients with acute coronary syndrome. However, prognostic performances of miRNAs in patients with acute heart failure (AHF) has received less attention.

**Methods:**

A test cohort of 294 patients with acute dyspnea (236 AHF and 58 non-AHF) and 44 patients with stable chronic heart failure (CHF), and an independent validation cohort of 711 AHF patients, were used. Admission levels of miR-1/-21/-23/-126/-423-5p were assessed in plasma samples.

**Results:**

In the test cohort, admission levels of miR-1 were lower in AHF and stable CHF patients compared to non-AHF patients (p = 0.0016). Levels of miR-126 and miR-423-5p were lower in AHF and in non-AHF patients compared to stable CHF patients (both p<0.001). Interestingly, admission levels of miR-423-5p were lower in patients who were re-admitted to the hospital in the year following the index hospitalization compared to patients who were not (p = 0.0001). Adjusted odds ratio [95% confidence interval] for one-year readmission was 0.70 [0.53–0.93] for miR-423-5p (p = 0.01). In the validation cohort, admission levels of miR-423-5p predicted 1-year mortality with an adjusted odds ratio [95% confidence interval] of 0.54 [0.36–0.82], p = 0.004. Patients within the lowest quartile of miR-423-5p were at high risk of long-term mortality (p = 0.02).

**Conclusions:**

In AHF patients, low circulating levels of miR-423-5p at presentation are associated with a poor long-term outcome. This study supports the value of miR-423-5p as a prognostic biomarker of AHF.

## Introduction

Acute heart failure (AHF) carries a high mortality risk and a high incidence of hospital readmission.[1] Prediction of outcome of AHF patients is a challenging task, even with recent prediction models involving multiple clinical predictor variables.[2] Various circulating peptides, including natriuretic peptides, have been shown to be of interest to improve the diagnosis and guidance of short and long term therapy in AHF patients but are poorly associated with outcome.[3] Recently, the 22 nucleotide-long non coding microRNAs (miRNAs) that are known to modulate gene expression have gained attention as potential biomarkers for personalized healthcare of patients with cardiac disease.[4, 5]

Since the discovery of miRNA presence in the bloodstream [6, 7], the potential of miRNAs to aid in disease management has been an active field of investigation. Their ability to diagnose patients with cardiovascular disease has been thoroughly addressed in small scale studies (reviewed in [8]). Subsequently, the diagnostic performance of circulating miRNAs was assessed in larger cohorts of patients with acute coronary syndrome [9] and acute myocardial infarction.[10, 11] In addition, some miRNAs were found to be prognostically valuable. [9, 12–15]

So far, the investigation of the biomarker value of circulating miRNAs has received less attention in the critically ill. Tijsen et al. identified a set of 6 miRNAs in HF patients among which miR-423-5p was strongly associated with AHF.[16] Fukushima et al. observed that circulating levels of miR-126 were negatively correlated with disease severity in patients with HF.[17] We found in AHF patients an elevation of circulating levels of cardiac-enriched miR-499.[18] It is worth mentioning that the results of these studies are limited by relatively small population size (< 40 cases).[16–18]

In the present study, we used two independent cohorts of AHF patients to assess association between cardiovascular miRNAs and long term outcome. In a first test cohort, we assessed the diagnostic and prognostic performance of 5 miRNAs (miR-1/-21/-23/-126/-423-5p). We selected these miRNAs because of their known association with cardiac hypertrophy (miR-1/-23), angiogenesis (miR-23/-126), apoptosis (miR-21/-23), and fibrosis (miR-21).[19] We also selected miR-423-5p which is up-regulated in failing hearts [20] and which circulating levels are associated with the clinical diagnosis of AHF.[16, 21] Lastly, we investigated the association between miR-423-5p and long term outcome in the validation cohort.

## Materials and Methods

### Study cohorts

The first population (= the test cohort) consisted of 294 patients admitted to the emergency department (ED) or the cardiac care unit (CCU) with the diagnosis of acute dyspnea, either related to AHF (n = 236) or to non-AHF (n = 58) according to ESC guidelines [22], during the entire study period (from February 2008 to June 2011). The diagnosis of AHF or non-AHF was performed by 2 senior physicians, experts in HF, using all clinical and biological parameters including plasma brain natriuretic peptide (BNP) level. BNP was measured within 4 h after admission in emergency department, on an Abbott Architect system (Abbott laboratories, Abbott Park, IL, USA). According to the study protocol, blood sampling was performed in ethylenediaminetetraacetic acid tubes within four hours after ED or CCU admission and plasma was immediately stored at– 80°C. Blood sampling was also performed and plasma stored in a subgroup of 64 patients, 5 days after admission for dyspnea. Demographic and biological parameters at admission along with hospital readmission and mortality data at one year of follow-up were recorded in AHF patients. In addition, plasma was also withdrawn from 44 chronic HF patients in stable condition (stable CHF) during an outpatient visit. The study was registered in clinical trials.gov under the identifier NCT01374880.

The second population (= the validation cohort) consisted of 711 AHF patients recruited from 2 Leicester hospitals between November, 2006-October, 2012. The diagnosis of AHF was made by 2 physicians, and all patients had evidence of fluid overload (pulmonary oedema or congestion) and oedema. Samples were obtained after 30 minutes bed rest, and plasma was stored at -80ᴼC until thawed for analysis. Endpoints such as death and rehospitalisation for heart failure were determined using local electronic patient records and the Office of National Statistics records. The study was approved by the Derbyshire Research Ethics Committee.

The present study was performed in accordance with the ethical guidelines of the declaration of Helsinki and all patients provided written consent.

### Measurement of circulating miRNAs

Total RNA was extracted from plasma samples using the mirVana PARIS kit (Ambion, Applied Biosystem, Lennik, Belgium) without enrichment for small RNAs. Spiked-in synthetic C. elegans miRNA controls (Qiagen, Venlo, The Netherlands) were added to plasma samples for correction of extraction efficiency. After DNase treatment, RNAs were reverse transcribed with the miScript reverse transcription kit (Qiagen). cDNA was diluted 10-fold before quantitative PCR with the miScript SYBR Green PCR kit (Qiagen). MicroRNA-specific miScript primer sets were obtained from Qiagen. A control without reverse transcriptase and a control without RNA were added to each PCR plate to ensure the absence of contaminating DNA and to check for non-specific amplification, respectively. Expression values were normalized using the mean Ct of the spiked-in controls and calculated with the formula: 2 exp (mean Ct spiked-in controls—Ct target miRNA). Additional technical details have previously been published.[10]

### Study objectives

The primary objective of the study was to assess prognostic properties of circulating miRNAs on long term, clinically relevant, events (death and/or readmission). The secondary objective was to assess diagnostic properties of circulating miRNA to discriminate between cardiac and non-cardiac origin of acute dyspnea.

### Statistical analysis

Continuous variables were expressed as median (interquartile range) and categorical variables as count (percentage). For all analyses, circulating levels of miRNAs were log-transformed. Levels of miRNAs were compared among several group of patients, namely AHF with or without history of CHF (respectively named acute decompensated HF—ADHF—or *de novo* AHF), acute dyspnea from non-cardiac origin (non-AHF) and stable CHF using first the Kruskal-Wallis test and then the pairwise Wilcoxon test using an adjusted p value according to the Holm method.[23]

The ability of miRNAs to discriminate dyspnea of cardiac from non-cardiac origin was studied using receiver operating characteristic (ROC) curve and the area under the ROC curve (AUC).[24, 25] Association of circulating levels of miRNAs with one-year outcome was assessed using the chi square test. To take into account a potential confounding effect of prognostic covariates, adjusted analyses were performed using logistic regression. The association between miR-423-5p and one-year mortality in the validation cohort was assessed using Kaplan Meier analyses.

Statistical analyses were performed using the R statistical platform (http://www.r-project.org/) and the SigmaPlot v12 software. A 2-sided p value < 0.05 was considered statistically significant.

## Results

### Patient characteristics

**Fig 1:**
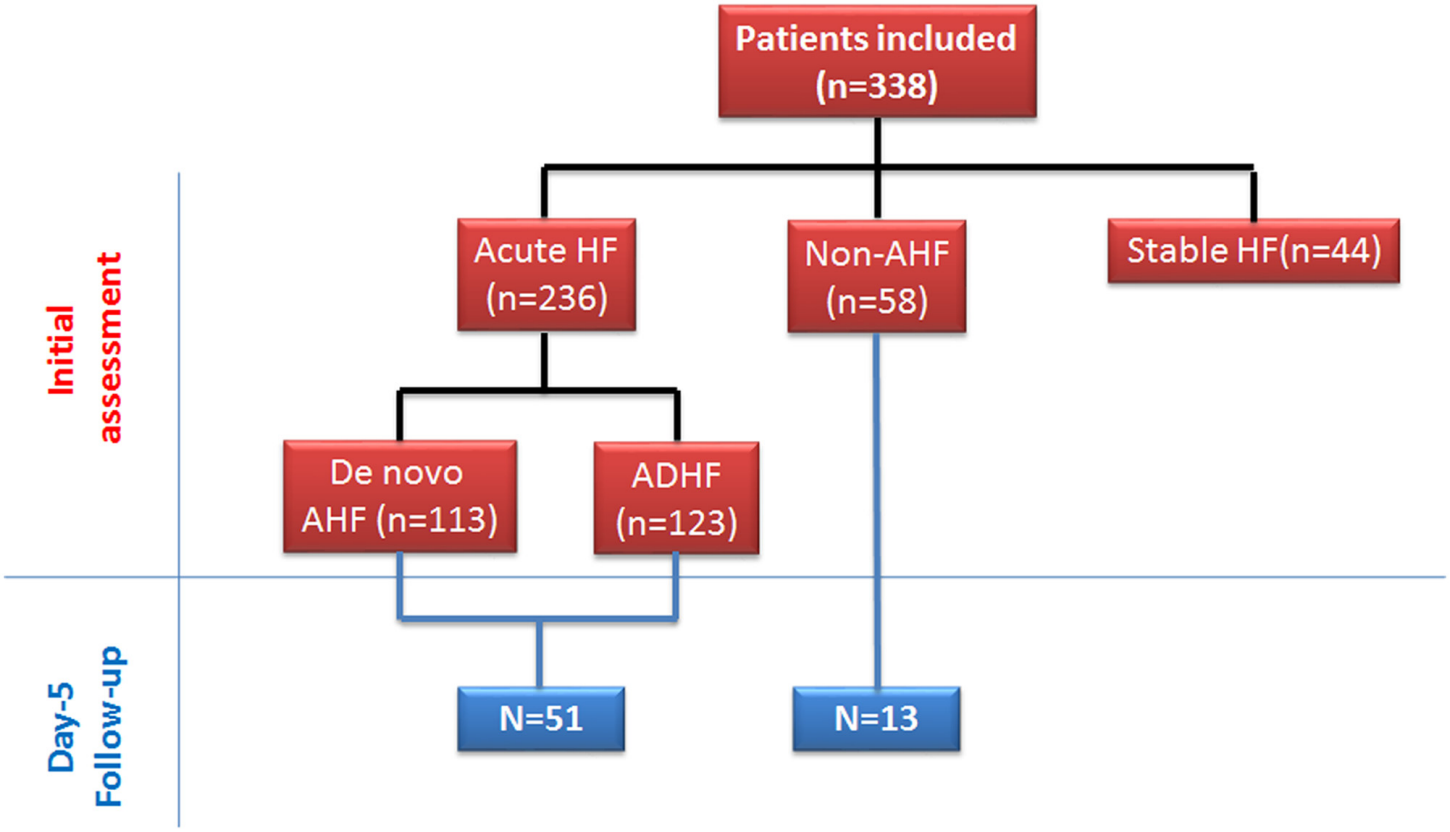
Flow chart of the study population.

The test cohort consisted of 294 patients admitted to the ED or CCU for acute dyspnea (Fig 1): 236 were diagnosed with AHF, and 58 with dyspnea of non-cardiac origin. In addition, 44 patients diagnosed with chronic stable heart failure (stable HF) were included. Table 1 shows that AHF patients had a high prevalence of history of cardiovascular disease and their median BNP plasma level on admission was 10-fold greater than in non-AHF patients.

**Table 1:** Patient characteristics of the test cohort.

	Acute dyspnea (n = 294)		Stable HF(n = 44)
	Acute HF (n = 236)	Non-AHF (n = 58)	
Age (years)	76 (65.5 to 84.5)	72.5 (62 to 79.75)	64 (52 to 71)
*Gender*			
Male	93 (39)	27 (47)	37 (86)
*Medical history*			
HF history	123 (52)	0 (0)	44 (100)
Atrial fibrillation	97 (41)	8 (14)	0 (0)
COPD/Asthma	30 (13)	38 (66)	5 (11)
Coronary artery disease	67 (28)	9 (16)	20 (45)
Diabetes mellitus	65 (27)	7 (12)	9 (20)
Chronic kidney disease	34 (14)	1 (2)	6 (14)
*Physical examination*			
Heart rate (bpm)	88 (71 to 107)	97 (84 to 110)	74 (63 to 90)
Systolic BP (mmHg)	135 (118 to 155)	134 (123 to 156)	109 (94.75 to 126)
Diastolic BP (mmHg)	78 (70 to 89)	76 (69.5 to 88)	70 (63 to 78)
*Echographic examination* LVEF (%)*	35 (25 to 56)		20 (20 to 30)
*Admission labs*			
BNP (pg/mL)	1242 (722 to 2394)	112 (38 to 157)	770 (430 to 1542)
Creatinine (μmol/L)	100 (75 to 139)	69 (60 to 80)	114 (92 to 150)
*Admission diagnosis (%)*			
Acutely decompensated HF	123 (52)	0 (0)	-
De novo acute HF	113 (48)	0 (0)	-
COPD/Asthma	0 (0)	48 (83)	-
Pulmonary embolism	0 (0)	3 (5)	-
Pneumonia	0 (0)	13 (22)	-
Other	0 (0)	13 (22)	-
*Treatment at admission*			
Beta-blockers	110 (37)	1 (2)	32 (73)
Statins	94 (32)	5 (9)	23 (52)
Antiplatelets	125 (42)	3 (5)	29 (66)
Calcium antagonists	69 (23)	3 (5)	0 (0)
Diuretics	150 (51)	6 (10)	27 (62)
ACE/ARB	156 (53)	11 (19)	32 (73)

ACE/ARB: angiotensin-converting enzyme inhibitors/angiotensin-receptor blockers; AHF: acute heart failure; BNP: B-type natriuretic peptide; BP: blood pressure; Bpm: beats per minute; COPD: chronic obstructive pulmonary disease; HF: heart failure; LVEF: left ventricular ejection fraction.

* LVEF was recorded by echocardiography during the index hospitalization in 80 patients.

The validation cohort consisted of 711 patients with AHF. Comparisons of clinical characteristics of the two patient populations revealed that patients of the validation cohort had a 3-fold lower readmission rate after 1 year compared to patients of the test cohort (Table 2). Also, patients of the validation cohort had more often a previous heart failure.

**Table 2:** Comparison of clinical characteristics of the test and validation cohorts.

	Test cohort (n = 236)	Validation cohort (n = 711)	p value
Age (year)	76 (65.5 to 84.5)	77 (68.6 to 83)	0.74
Gender (male)	143 (60.6)	456 (64.2)	0.32
Previous HF	119 (50.4)	249 (35.1)	**<0.0001**
SBP	135 (118 to 155)	134 (117 to 150.8)	0.24
DBP	78 (70 to 89)	75 (65 to 85)	**0.03**
Creatinin	100 (75 to 139)	110 (89.8 to 139)	**0.004**
Sodium	138 (135 to 141)	138 (135 to 141)	0.15
Atrial fibrillation	75 (31.8)	183 (34.5)	0.47
Hemoglobin	12.4 (11.2 to 13.4)	12.4 (10.8 to 13.8)	0.97
LVEF (%)	35 (25 to 56.2)	35 (26 to 47)	0.56
One-year death rate	41 (17.4)	154 (21.7)	0.16
One-year readmission rate	176 (74.6)	119 (25.6)	**<0.0001**
One-year combined outcome	196 (83.1)	248 (44.6)	**<0.0001**

Continuous variables are presented as median (range) and categorical variables are presented as number (frequency). DBP: diastolic blood pressure; HF: heart failure; LVEF: left ventricular ejection fraction; SDB: systolic blood pressure.

Full datasets of the test and validation cohorts are available in S1 and S2 Tables.

### Time-course of circulating miRNAs in the test cohort

**Fig 2:**
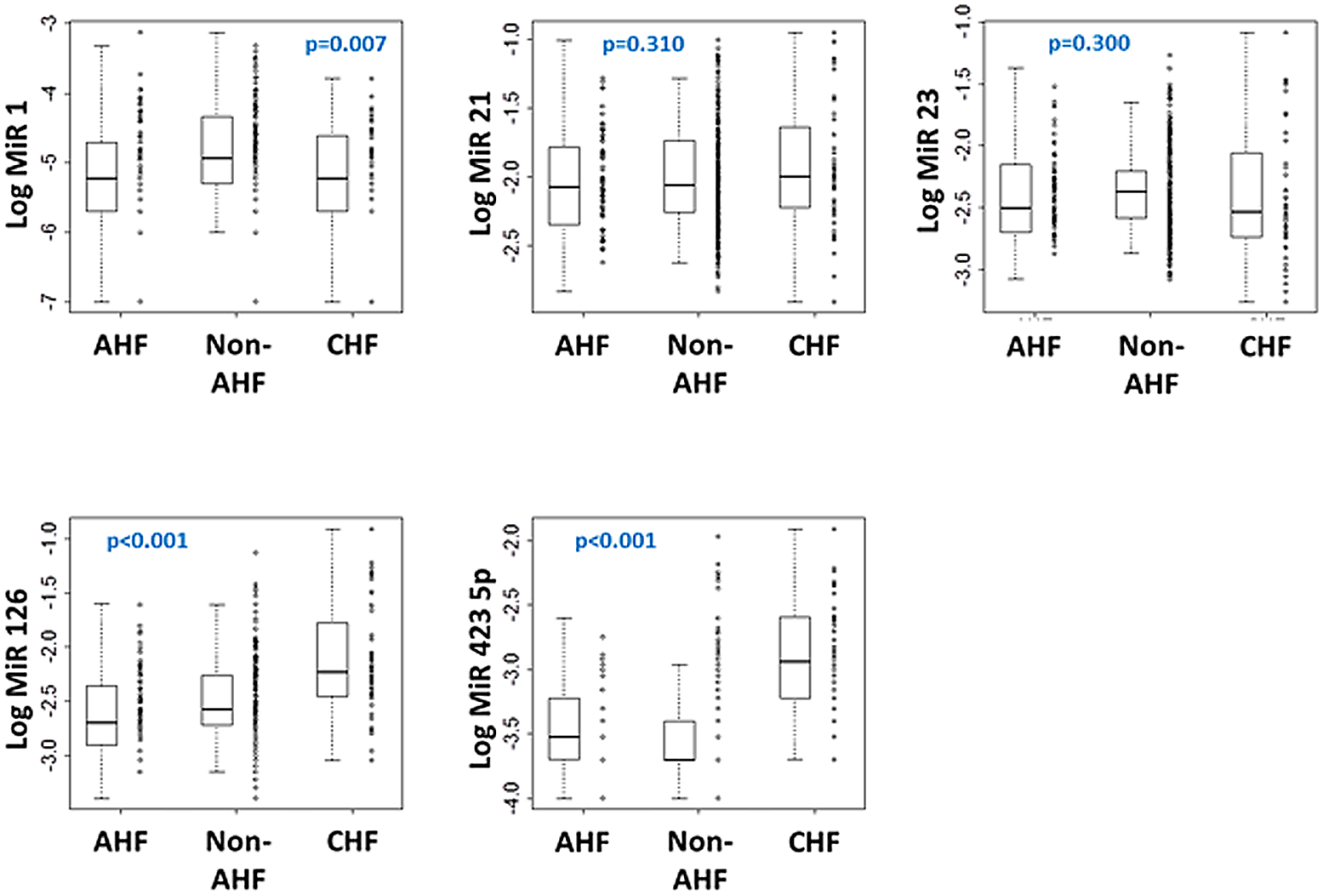
Admission levels of miRNAs. Levels of 5 miRNAs were assessed in plasma samples obtained at admission in 236 AHF patients 58 non-AHF patients, and 44 CHF patients. Between-group comparisons were performed and corresponding p values are indicated.

The plasma levels at admission of five miRNAs known to regulate multiple pathways activated in the failing heart were compared among AHF, non-AHF and stable CHF groups (Fig 2). Ranges of raw Ct values for each miRNA are presented in S3 Table. No non-specific amplification could be detected in the present study. Circulating levels of miR-1 were low in AHF or stable CHF compared to non-AHF (p = 0.0016). Levels of miR-126 and miR-423-5p were both lower in AHF and non-AHF on admission compared to stable CHF patients (both p<0.001). Levels of miR-21 and miR-23 were similar among the three patient groups. Levels of the 5 miRNAs studied were comparable between *de novo* AHF and ADHF (data not shown). Furthermore, admission levels of all 5 miRNAs displayed poor diagnostic performance for AHF (AUCs below 0.70) compared to BNP (AUC = 0.97) (S1 Fig). Combination of two or three miRNAs did not provide a clinically relevant diagnostic value (AUCs below 0.75; not shown).

**Fig 3:**
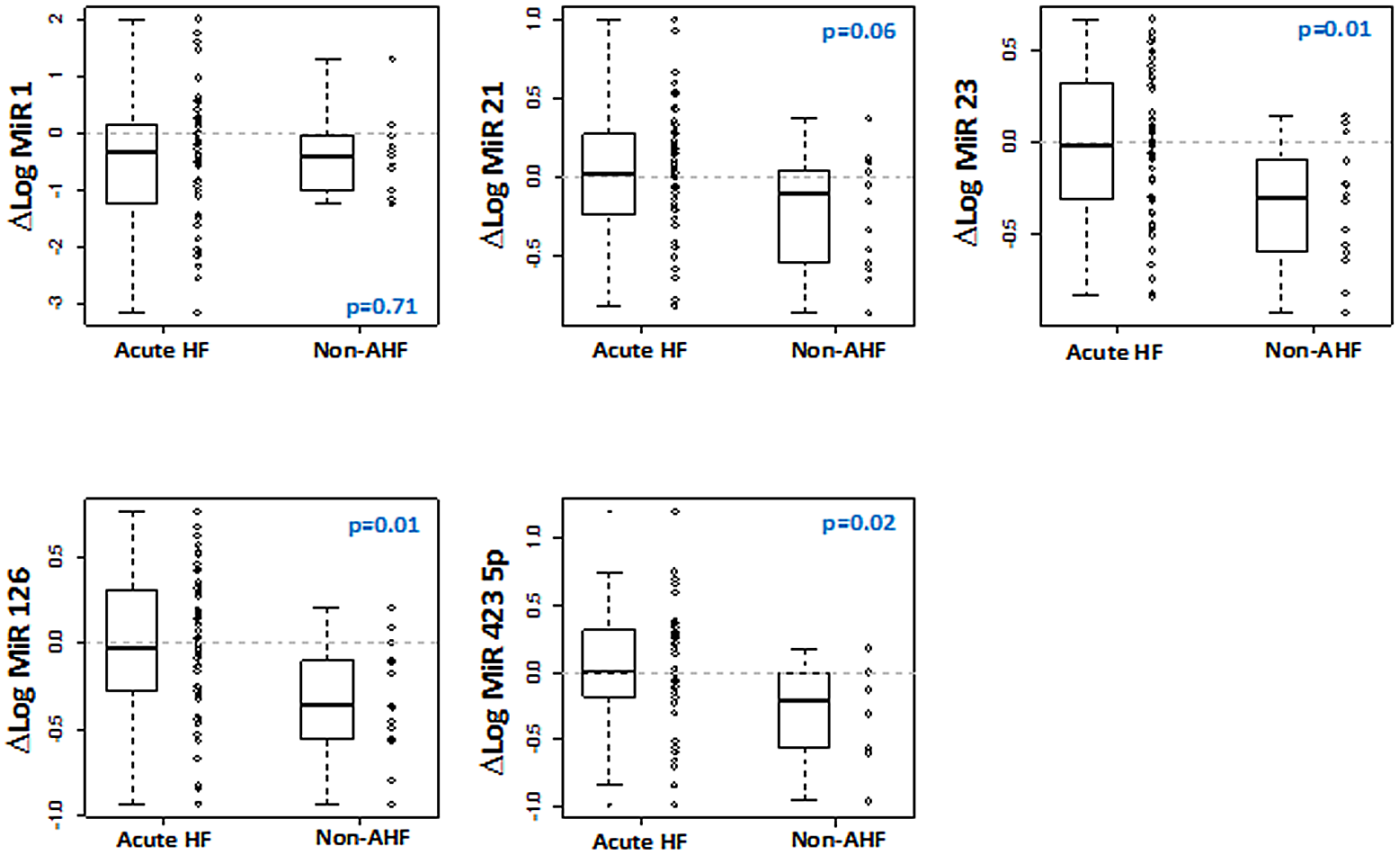
Time-course of plasma miRNA levels. The change between miRNA levels at admission and day 5 was calculated and compared between AHF patients (n = 51) and non-AHF patients (n = 13). P values for comparison between levels at admission and at day 5 are indicated for non-AHF. For AHF patients, all p values are > 0.05.

The kinetic of miRNA plasma levels was determined by calculating the change between admission and day 5 in 64 patients with acute dyspnea in the test cohort (51 AHF and 13 non-AHF; Fig 3). Overall, while the plasma levels of the 5 miRNAs remained unchanged in patients with AHF over the 5 days following admission, patients with non-AHF showed a decrease in the levels of miR-23, miR-126 and miR-423-5p over the same period.

### Circulating miRNAs and one-year outcome in AHF patients of the test cohort

During the year following admission, 176/236 (75%) AHF patients had a hospital readmission, mostly for cardiovascular disease 156/176 (89%). Among the 236 AHF patients, 41 (17%) died during the year following admission. As shown in Table 3, plasma levels of miR-21, miR-126 and miR-423-5p at admission were lower in patients who were readmitted in the year following the index hospitalization compared to patients who were not. However, plasma levels of miRNAs were similar between patients who survived and patients who died within the one-year of follow-up. Of note, LVEF, sodium, BNP, creatinine, proteins, or hemoglobin were unaffected by re-admission or survival.

**Table 3:** MiRNA levels according to one-year outcome.

**A**			
**Hospital Readmission**	**No readmission (n = 60)**	**Readmission (n = 176)** *	**p value**
BNP (pg/mL)	1358 (834 to 2472)	1151 (707 to 2375)	0.3
Creatinine (μmol/L)	97 (75.5 to 145)	104 (75 to 137)	0.77
LVEF (%)^#^	28 (24 to 31)	39 (25 to 60)	0.16
Plasma sodium (mmol/L)	138 (134 to 140.5)	138 (135 to 141)	0.74
Proteins (g/L)	69.5 (64.25 to 73)	71 (66 to 75)	0.32
Hemoglobin (g/dL)	12.2 (11.1 to 13.35)	12.4 (11.275 to 13.4)	0.79
miR-1 (10^6^)	6.5 (2.75 to 19.75)	6 (2 to 20)	0.83
**miR-21 (10** ^**3**^ **)**	**11.5 (6.2 to 24.9)**	**7.4 (4.2 to 15.2)**	**0.013**
miR-23 (10^3^)	4.2 (2.0 to 10.0)	3.1 (2.0 to 6.4)	0.17
**miR-126 (10** ^**3**^ **)**	**2.8 (1.6 to 6.2)**	**1.85 (1.2 to 3.6)**	**0.011**
**miR-423 5p (10** ^**3**^ **)**	**0.5 (0.3 to 1.0)**	**0.2 (0.1 to 0.5)**	**0.0001**
**B**			
**Death**	**Survived (n = 195)**	**Died (n = 41)**	**p value**
BNP (pg/mL)	1188 (729.75 to 2332.5)	1338 (646.5 to 2675)	0.87
Creatinine (μmol/L)	97 (75 to 136)	114 (85 to 161)	0.097
LVEF (%)^#^	35 (25 to 57.5)	35 (20 to 45)	0.60
Plasma sodium (mmol/L)	138 (135 to 140)	137 (133 to 141)	0.64
Proteins (g/L)	71 (66 to 75)	70 (64 to 74)	0.48
Hemoglobin (g/dL)	12.4 (11.3 to 13.4)	12.2 (11.1 to 13.2)	0.48
miR-1 (10−^6^)	6 (2 to 20)	7 (3 to 17)	0.56
miR-21 (10−^3^)	8.373 (4.518 to 15.72)	9.0 (4.9 to 16.5)	0.79
miR-23 (10−^3^)	3.058 (1.987 to 6.723)	4.0 (2.7 to 9.0)	0.18
miR-126 (10−^3^)	2 (1.3 to 4.4)	2.2 (1.2 to 4.2)	0.91
miR-423-5p (10−^3^)	0.3 (0.2 to 0.6)	0.3 (0.2 to 0.5)	0.61

BNP: B-type natriuretic peptide. LVEF: left ventricular ejection fraction.

* 156/176 (89%) patients were readmitted for cardiovascular causes.

^#^ LVEF was recorded in 80 patients. MicroRNA expression values were calculated with the formula 2 exp (mean Ct spiked-in controls—Ct target miRNA).

**Fig 4:**
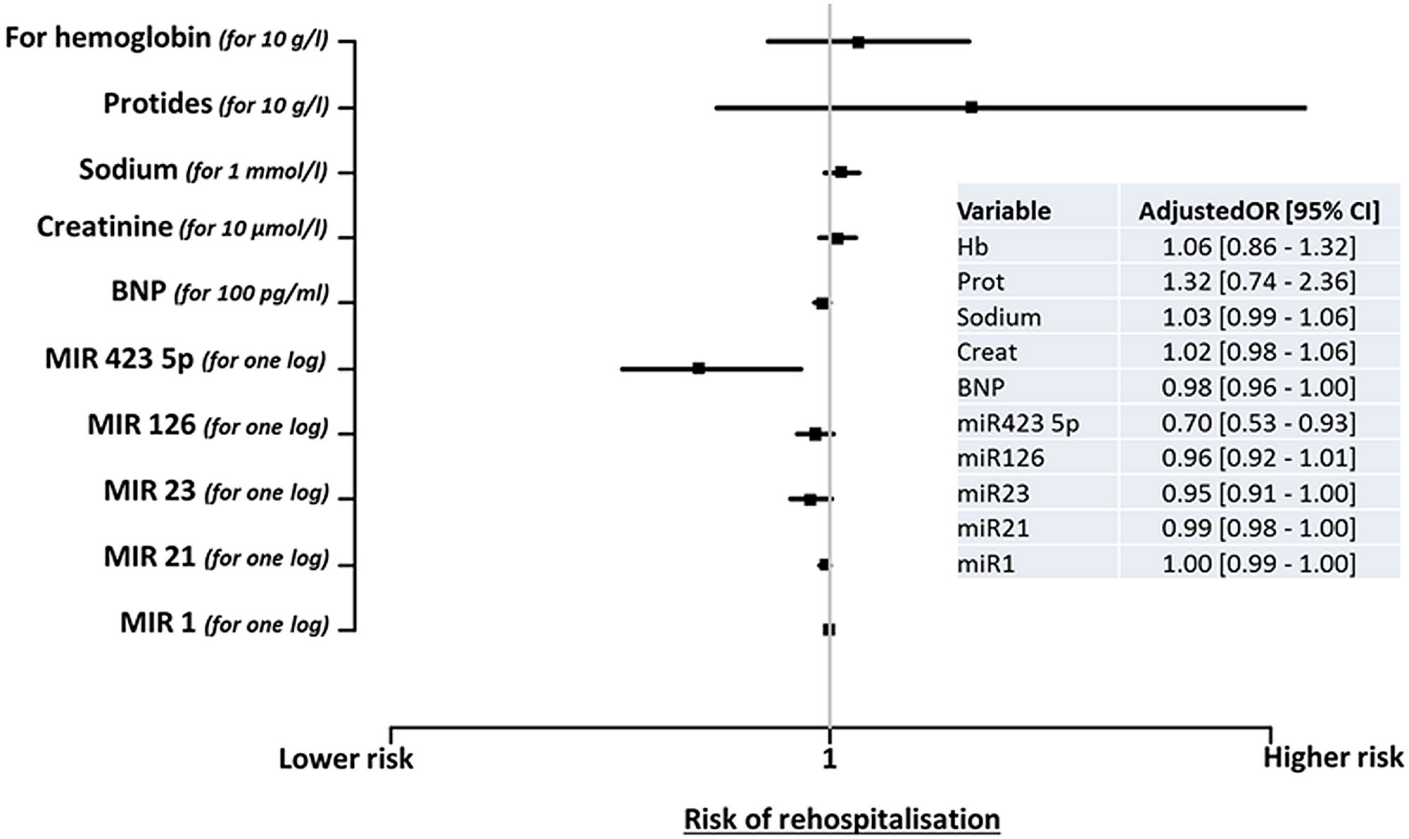
Association between miRNA levels and hospital readmission using multivariable analyses. Odds ratios (OR) [95% confidence interval] are shown for biological parameters, including miRNAs for the risk of hospital readmission during the first year following initial presentation. ORs have been adjusted for age, gender, heart rate, systolic and diastolic blood pressure, history of atrial fibrillation and of HF, LVEF, BNP, sodium, creatinine, proteins and hemoglobin.

Using multivariable analyses including the 5 studied miRNAs, the odds ratios (OR) [95% confidence interval] for hospital readmission, adjusted for age, gender, heart rate, systolic and diastolic blood pressure, history of atrial fibrillation and of heart failure, left ventricular ejection fraction, plasma levels of BNP, sodium, creatinine, proteins and hemoglobin, was only significant for miR-423-5p (0.70 [0.53–0.93], p = 0.01) (Fig 4). Of note, left ventricular ejection fraction was also associated with the risk of hospital readmission (OR 2.25 [1.16–4.34]). Admission levels of miRNAs were not associated with mortality of AHF patients within the year following the index hospitalization (not shown).

### Circulating miR-423-5p and outcome in the validation cohort

**Fig 5:**
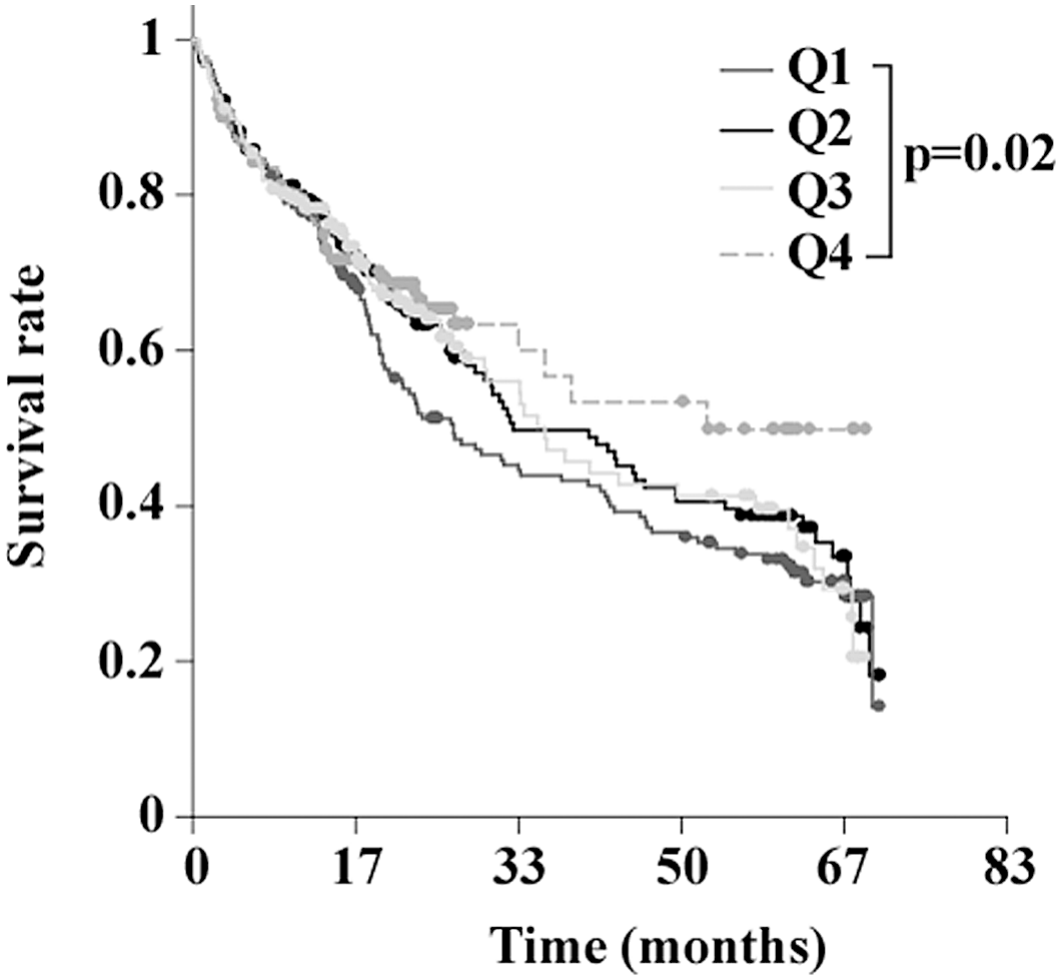
Association between miR-423-5p levels and mortality in the validation cohort (n = 711). The cohort was divided in quartiles of miR-423-5p values and Kaplan-Meier analysis was performed with calculation of the log rank to assess statistical significance. Q denotes quartile and p values from log rank test are displayed.

Levels of miR-423-5p measured at admission in the 711 AHF patients of the validation cohort were comparable to the test cohort: 0.03 [0.0006–1.12] vs. 0.03 [0.01–1.75] (median [range]), respectively, p = 0.97. Median follow-up time was 20 months, during which 119 patients were readmitted and 154 patients died. In multivariable analyses, miR-423-5p was not a significant predictor of 1-year readmission in this cohort (OR 0.82 [0.47–1.42], p = 0.48). However, miR-423-5p significantly predicted mortality (OR 0.54 [0.36–0.82], p = 0.004) (Table 4). Kaplan-Meier analysis revealed that patients within the lowest quartile of miR-423-5p levels had a higher risk of mortality compared to patients with low levels of miR-423-5p (p = 0.02, Fig 5). This association was evident from 2 years after the initial event.

**Table 4:** Prediction of death by miR-423-5p in the validation cohort.

Variable	Odds ratio	95% CI	P value
Age	1.05	1.03–1.07	**<0.001**
Sex	0.79	0.53–1.19	0.27
**History of**			
Heart failure	1.68	1.12–2.50	**0.01**
Ischemic heart disease	3.25	2.10–5.03	**<0.001**
Hypertension	0.61	0.41–0.89	**0.01**
Renal failure	2.18	1.30–3.67	**0.003**
**Admission parameters**			
Heart rate	1.00	0.99–1.01	0.92
Systolic blood pressure	0.99	0.99–1.00	0.16
Respiratory rate	1.04	1.00–1.07	**0.03**
Urea	1.04	0.98–1.10	0.18
Creatinine	1.00	0.99–1.01	0.78
Sodium	0.98	0.95–1.01	0.20
Log miR-423-5p	0.54	0.36–0.82	**0.004**

Multivariable analyses with logistic regression to predict mortality in the 711 patients of the validation cohort. CI: confidence interval.

## Discussion

The main finding of the present study is that low plasma levels of miR-423-5p are associated with a poor outcome of patients with AHF. This finding was confirmed in two independent patient cohorts.

The 5 miRNAs investigated in the present study are all related to HF, either through the regulation of critical pathways involved in the development and progression of HF (miR-1/-21/-23/-126), or because of their regulation in the failing heart (miR-423-5p). Although circulating levels of some of these miRNAs were previously shown to be associated with the diagnosis of CHF [16, 17], both at admission and at follow-up, none of them provided a diagnostic value similar to BNP for AHF, in the present study. Of note, the robust diagnostic value of BNP for AHF (AUC 0.97) can be explained by the fact that it was available to physicians at the time of adjudication. In particular, miR-423-5p, which was reported to discriminate HF cases from healthy controls in the studies by Tijsen [16] and Goren [21], failed to provide a diagnostic value for AHF in our study (AUC = 0.61), even though its plasma level was lower in AHF patients compared to stable CHF patients. This is likely related to the use of healthy controls in the studies by Tijsen [16] and Goren [21], compared to the use of all dyspneic patients in our study. Consistently with our findings, Ellis and colleagues [26] recently showed that miR-423-5p was not significantly associated with HF diagnosis in ROC analysis (AUC 0.58). However, in this study, miR-423-5p increased the diagnostic value of N-terminal pro-BNP (AUC 0.90 for N-terminal pro-BNP and 0.93 for N-terminal pro-BNP with miR-423-5p). This incremental value, although very modest (+3.2%), was statistically significant (P = 0.03). In our study, miR-423-5p provided no additive value to BNP (not shown). In the recent study of Vogel et al., miR-423-5p was not significant dysregulated in whole peripheral blood of HF patients with reduced EF [27]. Whether miR-423-5p provides a clinically relevant incremental diagnostic value to BNP is still uncertain and deserves further testing in independent cohorts. In Ellis [26] and our study, miR-423-5p was down-regulated while it was mostly elevated in other studies [16, 21]. This may be explained by differences in disease phenotype but also by the regulation of circulating levels of miR-423-5p from non-cardiac cause [28, 29]. As such, miR-423-5p may reflect the pathological status of not only the heart but also other organs.

Serial samples allowed to investigate the evolution of circulating levels of miRNAs within the 5 days following admission. Significant differences in levels of miR-23, miR-126 and miR-423-5p between AHF and non-AHF patients were observed, suggesting that the circulating levels of these miRNAs are differently regulated in AHF and non-AHF patients. The mechanisms beyond this difference remain to be elucidated.

Hospital readmission is still a major issue in the follow-up of AHF patients. One patient out of 4 is readmitted within 30 days and one half within 6 months [30, 31]. In our test cohort, one-year all-cause readmission rate was 74.6%. By contrast, readmission rate was much lower in the validation cohort; this might be related to various causes including lower incidence of previous history of HF though similar levels of miRNAs were measured in de novo and known history of CHF. This issue needs further investigation.

Moderate diagnostic value of miRNAs does not preclude strong prognostic properties. Indeed, in the MOCA study, soluble ST2 or mid-regional adrenomedullin have lower diagnostic values though stronger prognostic properties than natriuretic peptides in AHF patients [3]. Similarly, we showed, in the present study, in the test cohort,that low plasma levels of miR-423-5p were able to identify AHF patients with high risk of hospital readmission, mostly for cardiovascular disease. Of note, in the test- cohort, biological parameters of congestion, namely BNP, proteins, hemoglobin or sodium, or parameters of heart function such as LVEF, were not associated with long-term outcome. This emphasizes the importance of our results linking miR-423-5p at admission with re-hospitalization in AHF patients. Indeed, early identification of these patients is clinically relevant since they may best benefit from post-discharge care, home-based follow-up and patient education.

In our validation cohort, we confirmed that low plasma levels of miR-423-5p are associated with a poor outcome, as indicated by a higher mortality rate. However, miR-423-5p levels did not predict hospital readmission in this cohort. This discrepancy between the test cohort and the validation cohort most probably reside from patient specificities. Indeed, patients from the test cohort had significantly higher readmission rate, showing that these patients had aggravated heart failure compared to patients from the validation cohort. The association between miR-423-5p and mortality in the validation cohort, as determined by Kaplan Meier analysis, is truly visible from 2 years after the initial event, showing that miR-423-5p is best suited to predict long-term fatal events. Of note, in our study, BNP measured at admission did not perform well to predict one-year outcome as previously described [3].

How miRNA determination can add to the value of existing plasma biomarkers deserves further investigation. Also, our data are in line with the concept that miR-423-5p is cardioprotective, as low levels are associated with a bad outcome; this needs to be experimentally investigated. Accordingly, the obvious clinical implication is that all efforts should be made to administer optimal CHF therapy to patients with low miR-423-5p both before discharge and early after discharge.

### Study limitations

We acknowledge that the present results suffer some limitations. Only 5 miRNAs were measured and other miRNAs of interest might have been measured. However, the choice of these miRNAs was rationalized and recent studies seem to confirm that these 5 miRNAs are relevant in the cardiovascular area. The time-course analysis of miRNA in dyspneic patients was performed in a subgroup of 64 patients; however, it showed marked differences between AHF and non-AHF patients. Our study is limited by an apparent lack of power which might explain the absence of predictive value of miR-423-5p for readmission in the validation cohort and for death in the test population. Also, RNA integrity in plasma samples was not verified before miRNA measurements. Finally, other statistical approaches could have been used to test difference among studied groups, such as a Bayesian approach. This could be done in further studies.

## Conclusions

Our study shows that admission levels of circulating miR-423-5p are markers of poor long term outcome, whether hospital readmission and/or long-term mortality, in AHF patients. These findings must be corroborated by further independent cohort studies. MiR-423-5p might be also investigated in more severe forms of AHF including cardiogenic shock where biomarkers are needed to help predicting failure of medical therapy and the need of ventricular device as bridge to therapy [32].

## Supporting Information

S1 Fig Comparison of diagnostic value of miRNAs and BNP for AHF. Plasma levels of miRNAs were measured at admission in 236 patients with AHF and 58 patients with non-AHF. ROC curves attest for the high diagnostic accuracy of BNP and low value of the 5 miRNAs studied.
(TIF)

S1 Table Dataset of the test cohort. (XLSX)

S2 Table Dataset of the validation cohort. (XLSX)

S3 Table Ranges of raw Ct values for each miRNA presented in Table 3 and in Fig 2. (XLSX)

## Abbreviations

ADHF: acute decompensated heart failure
AHF: acute heart failure
AUC: area under the curve
BNP: brain natriuretic peptide
CCU: cardiac care unit
CHF: chronic heart failure
CI: confidence interval
ED: emergency department
HF: heart failure
miRNA: microRNA
OR: odds ratio
ROC: receiver operating characteristic
