# Supplementary figures and images for: Circulating microRNAs and Outcome in Patients with Acute Heart Failure
Source: PLoS One. 2015 Nov 18;10(11):e0142237. doi: 10.1371/journal.pone.0142237 (PMC4651494; doi:10.1371/journal.pone.0142237)

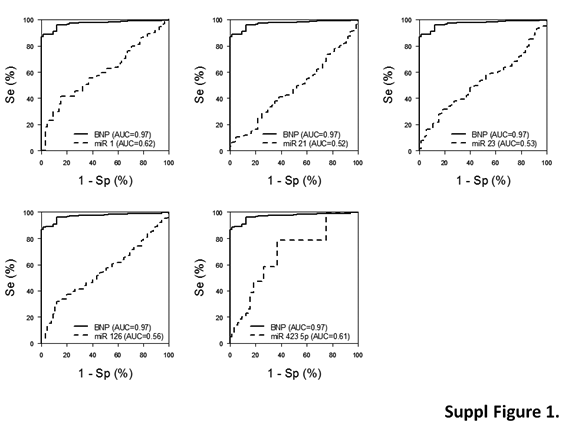

Supplement: S1 Fig — Plasma levels of miRNAs were measured at admission in 236 patients with AHF and 58 patients with non-AHF. ROC curves attest for the high diagnostic accuracy of BNP and low value of the 5 miRNAs studied. (TIF) [file pone.0142237.s001.tif]
